# The LARK RNA-Binding Protein Selectively Regulates the Circadian Eclosion Rhythm by Controlling E74 Protein Expression

**DOI:** 10.1371/journal.pone.0001107

**Published:** 2007-10-31

**Authors:** Yanmei Huang, Ginka Genova, Mary Roberts, F. Rob Jackson

**Affiliations:** Department of Neuroscience, Center for Neuroscience Research, Tufts University School of Medicine, Boston, Massachusetts, United States of America; Victor Chang Cardiac Research Institute, Australia

## Abstract

Despite substantial progress in defining central components of the circadian pacemaker, the output pathways coupling the clock to rhythmic physiological events remain elusive. We previously showed that LARK is a Drosophila RNA-binding protein which functions downstream of the clock to mediate behavioral outputs. To better understand the roles of LARK in the circadian system, we sought to identify RNA molecules associated with it, in vivo, using a three-part strategy to (1) capture RNA ligands by immunoprecipitation, (2) visualize the captured RNAs using whole-genome microarrays, and (3) identify functionally relevant targets through genetic screens. We found that LARK is associated with a large number of RNAs, in vivo, consistent with its broad expression pattern. Overexpression of LARK increases protein abundance for certain targets without affecting RNA level, suggesting a translational regulatory role for the RNA-binding protein. Phenotypic screens of target-gene mutants have identified several with rhythm-specific circadian defects, indicative of effects on clock output pathways. In particular, a hypomorphic mutation in the E74 gene, *E74^BG01805^*, was found to confer an early-eclosion phenotype reminiscent of that displayed by a mutant with decreased LARK gene dosage. Molecular analyses demonstrate that E74A protein shows diurnal changes in abundance, similar to LARK. In addition, the *E74^BG01805^* allele enhances the lethal phenotype associated with a *lark* null mutation, whereas overexpression of LARK suppresses the early eclosion phenotype of *E74^BG01805^*, consistent with the idea that E74 is a target, *in vivo*. Our results suggest a model wherein LARK mediates the transfer of temporal information from the molecular oscillator to different output pathways by interacting with distinct RNA targets.

## Introduction

The circadian system of living organisms consists of three formal components: a molecular oscillator that generates and maintains circa 24-hour rhythms, input pathways that synchronize (i.e., entrain) the intrinsic pacemaker to environmental cues, and output pathways that couple the clock to individual physiological processes. In recent years, there has been significant progress towards understanding clock entrainment mechanisms and the molecular [1], [2] and cellular [3], [4] elements of neural pacemakers; however, clock output mechanisms are still poorly understood [5]. Peptidergic clock output factors [6]–[9] have been identified in mammals and insects, but neither the intracellular signaling pathways regulating rhythmic peptide release nor the target cells of such peptides have been well delineated [5], [10]–[14]. Initial approaches to identify clock output pathways utilized subtractive hybridization procedures in *Neurospora* to define clock-controlled genes (CCGs, [10], [15]). More recent approaches have utilized microarray-based, genome-wide expression profiling studies to define CCGs, and these have revealed hundreds of genes that are transcribed in a circadian manner [16]–[22]. However, there is great variation among the microarray-based studies with regard to identified CCGs. In addition, such an approach is inherently limited to the identification of “cycling RNAs” and does not define clock-controlled changes in RNA translation or protein stability events. Importantly, recent studies found that approximately 20% of soluble proteins assayed in mouse liver extracts are under circadian control, but at least half of the corresponding RNAs encoding these proteins do not cycle in abundance [23], consistent with previous results suggesting an important role for post-transcriptional regulation in circadian control.

Several RNA-binding proteins with presumed post-transcriptional roles in the circadian system have been defined [24]–[27]. A Drosophila RNA-binding protein known as LARK exhibits circadian changes in abundance and is thought to function downstream of the molecular oscillator to mediate behavioral outputs [25], [28], [29]. LARK is in the RNA Recognition Motif (RRM) class of RNA binding proteins, and more specifically defines a class of RRM proteins containing a retroviral-type zinc finger [24]. Members of the RRM protein family are known to function in many different post-transcriptional regulatory processes, including the control of RNA splicing, intracellular transport, stability and translation [30]. In order to better understand the roles of LARK in the Drosophila circadian system, we have utilized a biochemical approach coupled with phenotypic screens of mutants to identify in vivo RNA targets of LARK. We report here that a large number of different RNAs are associated with LARK, in vivo, including several with known circadian functions. As proof of principle for our approach, we present an analysis of one target-expressing gene–Eip74EF (aka E74)– and show that it has an important role in the circadian control of population eclosion.

## Results

### LARK is associated with many different RNAs in the Drosophila central nervous system

We employed a “Ribonomics” approach [31] to identify RNAs that are associated with LARK *in vivo*. Our techniques were based on those of Tenenbaum et al (2002) but they differed from the previously published methods in several ways (see Materials and Methods). In our studies, LARK-containing ribonucleoprotein complexes (LARK-RNPs) were precipitated from lysates of hand-dissected pharate adult brains using an affinity-purified anti-LARK antibody ([25]; see Materials and Methods). A portion of each lysate was saved prior to immunoprecipitations (IPs) in order to measure the relative abundance of transcripts in a total RNA sample. RNAs extracted from the immunoprecipitated (IP) and total RNA samples were labeled and hybridized to Drosophila whole-genome gene microarrays; signal intensities for individual genes were compared between samples to identify those RNAs that were enriched by immunoprecipitation (relative to their abundances in total RNA). RNAs that were selectively enriched in the IP samples were considered to be potential targets of the RNA-binding protein. Microarray data analysis and the criteria for identification of LARK targets are discussed in greater detail in the Materials and Methods.

We identified 144 and 151 putative LARK targets, respectively, in two independent experiments (Table S1), with 79 targets in common between the experiments (see GEO series accession numbers GSE6420 and GSE6418). We note that RNA samples employed in several control experiments, in which the LARK antibody was not present (beads only), did not reveal selective enrichment of specific RNA molecules (data not shown), indicative of specific binding. It is worth mentioning that a mammalian homolog of LARK, the mouse RBM4 protein (mLARK), has six known targets [32] and two of the mLARK targets, calmodulin and flotillin, were also identified in our experiments as targets of fly LARK (dLARK). Two other targets of mLARK, RhoC and RpL27A, have counterparts in the dLARK target collection: the dLARK targets sar1 and R both encode GTPases with similarity to RhoC, whereas CG9354 and CG9282 encode components of the large subunit of the ribosome (RpL34 and RpL24, respectively).

### LARK target RNAs share common sequence and organizational features

**Figure 1:**
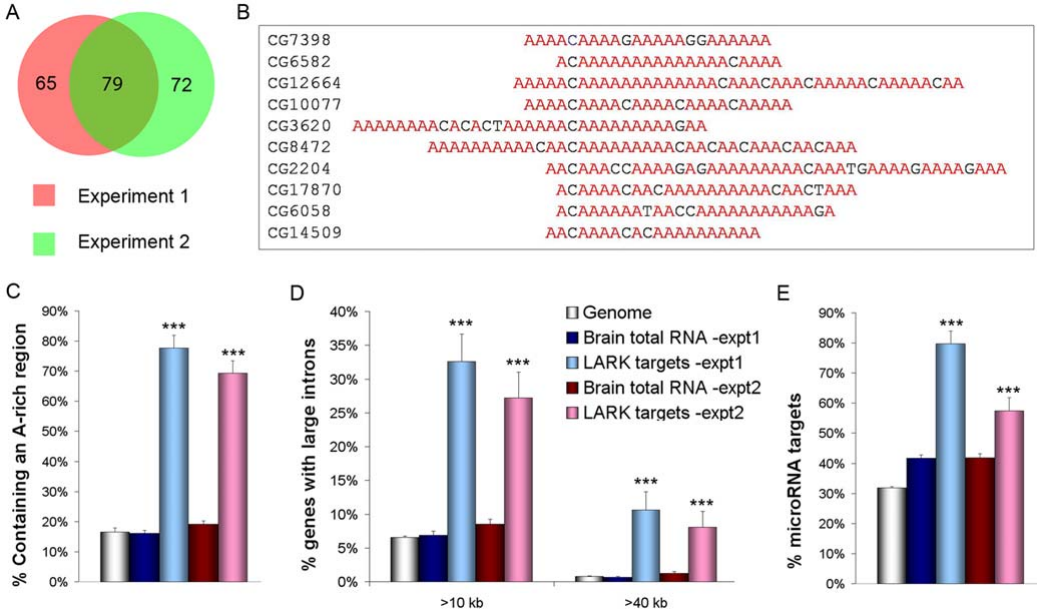
Identification of potential LARK target RNAs from the pharate adult fly brain. A) Number of putative target RNAs identified in two independent immunoprecipitation experiments. The overlapping area of the Venn diagram represents the number of targets common to both experiments. B) 3′UTR sequences of LARK target RNAs that contain A-rich elements with one or more “ACAAA” motifs. C–E) Comparison of the frequencies of particular sequence features in the entire Drosophila genome, in brain RNAs (those detected in brain total RNA), and0 in LARK targets. C) Frequencies of targets containing an A-rich region. D) Frequencies of target genes containing a large intron; E) Frequencies of targets containing miRNA binding sites. *** p<0.001 based on Chi-square test for equality of distributions. Error bars represent standard error of the binomial distribution.

Sequence analysis of the putative LARK targets identified in either immunoprecipitation experiment revealed that many of them contain one or several A-rich regions within the 3′ UTR. For the targets from experiment one that contain an annotated 3′UTR longer than 15-nucleotides, 77.7% (73 out of 94) contain an A-rich region. Similar results (63.0%, or 80 out of 127) were observed in experiment two (Figure 1C). These blocks of sequence were observed to be at least 15 bases in length with 75% of the sequence containing A residues; in many cases, there were intervening C or G bases within the A-tract. In addition, the A tracts often contain one or more copies of an “ACAAA” motif (Figure 1B). These A-rich tracts were found to be located within the 3′ UTR but not in any particular position relative to the poly(A) recognition signal (AATAAA). As shown in Figure 1C, such A-rich blocks of sequence are not present at a high frequency in the 3′UTRs of either brain total RNAs (16.2%, 320/1975 for experiment one and 19.2%, 289/1506 for experiment two) or RNAs randomly sampled from the genome (16.7%, 127/765). While we do not know the function of these A-rich tracts, we note that a similar A-rich sequence is found in a circadian target of the mouse mLARK protein [33].

An interesting organizational feature of the transcription units expressing LARK target RNAs is the presence of at least one large intron. Our survey found that 6.5% of the intron-containing genes in the entire Drosophila genome contain an intron larger than 10 kb; a similar proportion (6.9% to 8.5%) is observed for genes expressed in the pharate adult brains (Figure 1D). In contrast, 32.6% and 27.2% of the intron-containing genes from the LARK target sets (from the two different experiments) contain at least one large intron (significantly different, p<0.001 based on Chi-square test for equality of distributions; Figure 1D).

It is also of interest that many of the LARK RNA ligands appear to be targets of microRNAs (miRNAs). miRNAs are small RNA molecules that repress mRNA translation in a sequence-specific manner [34]. It is not surprising that neurally expressed RNAs are targeted more frequently by miRNAs; such RNAs are often present in locations (axons, dendrites) distant from the nucleus and regulated acutely by post-transcriptional mechanisms [35]. However, LARK target RNAs contain miRNA binding sites at a significantly higher frequency than brain total RNA samples. A search of the Drosophila miRNA database [36], for example, indicated that 79.8% of the LARK targets from experiment one contain binding sites for miRNAs, whereas only 41.8% of mRNAs detected in the total RNA sample contain such sites. Similarly, miRNA binding sites are present at increased frequency in the LARK targets identified in experiment two (Figure 1E).

### LARK overexpression increases the abundance of certain target-encoded proteins

To determine whether a change in LARK abundance altered target RNA levels, we queried whole genome microarrays with total RNA samples from *elav-gal4/+; UAS-lark*/+ flies (pan-neuronally overexpressing LARK) and *elav-gal4*/+; +/+ (control) flies, respectively. We found that for experiment one, only 14.6% (21/144) target RNAs show greater than a two-fold change (increased or decreased) in abundance when LARK is overexpressed. A similar result (16.6% or 25/151) was observed for the targets identified in experiment two. Thus, for the majority of the targets, there is not a significant effect of LARK overexpression on RNA abundance, as assayed by microarray analysis. We note that there were other RNAs, besides potential targets, which displayed significant changes in abundance in response to LARK overexpression (GEO accession number: GSE6420); these may be expressed from genes downstream of LARK. Presumably they are in some way indirectly regulated by LARK. They have not been further studied.

**Figure 2:**
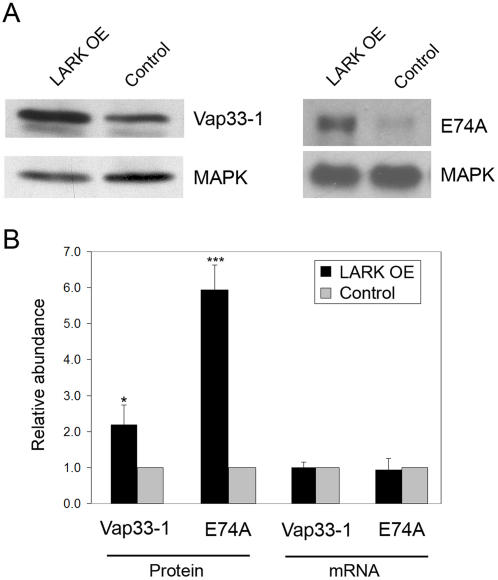
Overexpression of LARK alters the abundance of proteins encoded by two target RNAs without affecting steady-state RNA abundance for the targets. A) Representative Western blots showing the abundance of proteins encoded by two LARK targets, Vap33-1 (left) and E74A (right), in flies overexpressing LARK (LARK OE) versus control flies. B) Quantification of relative protein amount and RNA level for the two target genes. RNA and protein samples were collected at ZT6, a time at which LARK abundance is high. Protein abundance was normalized to that of MAP Kinase (MAPK). RNA levels were normalized to *Ribosomal protein 49* (*rp49*). * n = 5, p<0.05; *** n = 7, p<0.001 based on Student's t-test. Error bar represents SEM.

We next asked whether LARK might influence the translation of particular target RNAs by performing immunoblotting experiments for two target-encoded proteins, Vap33-1 and E74A, for which antibodies were available. As shown in Figure 2, we found that the overexpression of LARK significantly increased the abundance of both proteins. However, whereas protein abundance increased for these targets, LARK overexpression had no significant effect on RNA abundance.

### Certain target-encoded proteins have circadian functions

Four of the LARK targets, *dunce* (*dnc*), *No Receptor Potential A* (*NorpA*), *flap wing* (*flw*) and *discs overgrown* (*dco,* a.k.a *double time, dbt*), have been shown in previous studies to be relevant for circadian functions [37]–[42]. In order to identify additional targets that might mediate circadian function(s) of LARK, we obtained and began screening available mutants for the 216 putative target genes; currently, mutants are available for 178 (82%) of this gene collection. We decided to screen mutants of all presumptive LARK targets, because of the concern that a phenotypic screen of only the 79 common genes (observed in both experiments) might miss *bona fide* target molecules. At present, we have assayed eclosion rhythms or locomotor activity rhythms for mutants of 69 genes or 14 genes, respectively. This ongoing screen has validated our biochemical genetic approach and identified several new mutants with defective eclosion or activity rhythms. These include mutants of the *Ecdysone-induced-protein 74EF* (*Eip74EF*, a.k.a. *E74*) gene. Interestingly, the E74 transcription unit displays features common to other potential LARK targets: it contains an A-rich element in the 3′UTR, the transcription unit contains a large ∼37-kb intron, and the 3′UTR contains binding sites for several miRNAs including miR-34, miR-9b, miR312, miR275, and miR-iab-4-5p. We note that *E74* was identified in only one of the two immunoprecipitation experiments, justifying the behavioral screen of mutants representing all presumptive target genes. We have characterized *E74* mutants in more detail as a proof of principle for our biochemical approach that identified LARK target RNAs.

### *E74*, a gene with a role in the circadian control of eclosion

**Figure 3:**
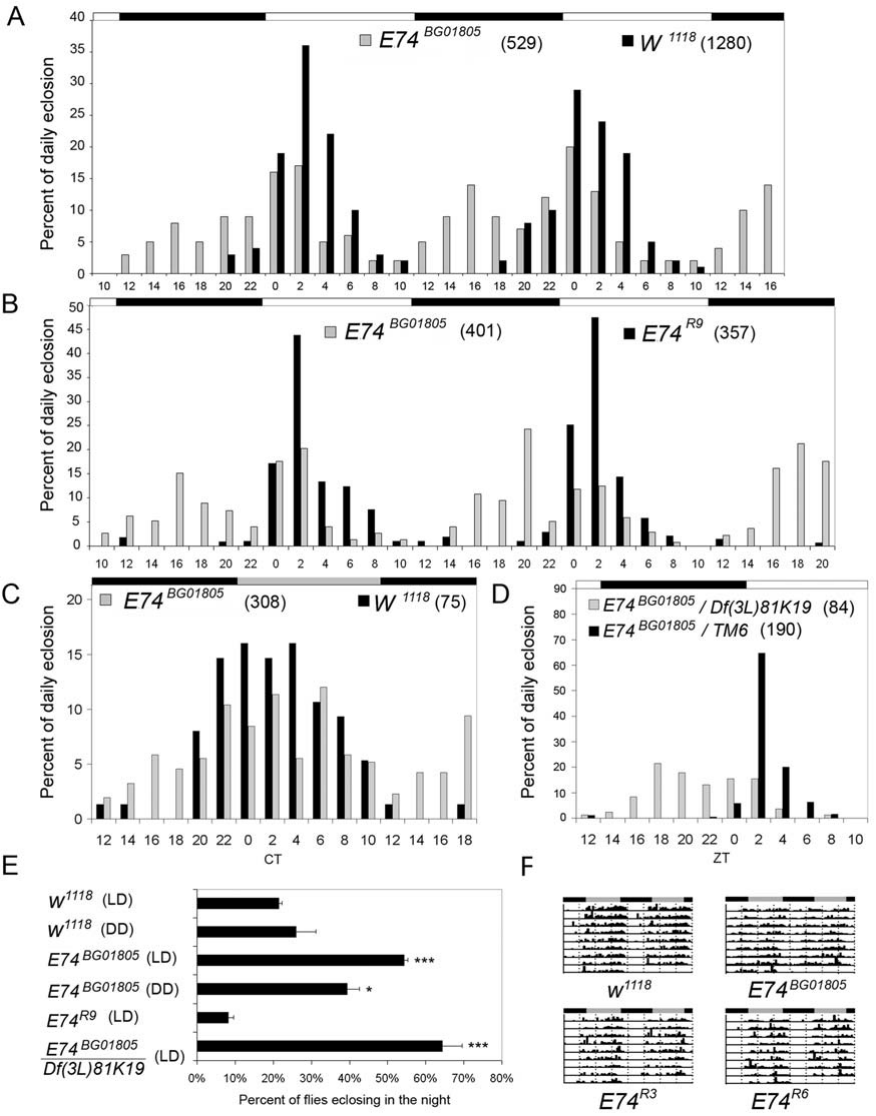
A hypomorphic mutation of the *E74* gene results in an early-eclosion phenotype but does not affect locomotor rhythmicity. A) LD eclosion profiles for control and *E74^BG01805^* homozygous populations. B) Eclosion profiles of *E74^BG01805^* mutant and revertant (*E74^R9^*) populations in LD. Excision of the P-element completely restored the wild-type eclosion profile. Similarly, reversion to the wild type was seen with two other independent P-element excision strains, *E74^R3^* and *E74^R6^* (not shown). C) Eclosion profiles for control and *E74^BG01805^* mutant populations in DD. D) Severe early-eclosion phenotype of transheterozygotes carrying *E74^BG01805^* in trans to a deletion of the gene [Df(3L)81K19]. E) Quantification of the percentage of flies eclosing between ZT10 and ZT22 for various genotypes. * P<0.05, *** P<0.0001 compared to *w^1118^*, based on Chi-sqare test for equality of distributions. F) Representative actograms for *w^1118^, E74^BG01805^*, and revertant males. In all panels of this figure, the light/dark schedule employed for entrainment is indicated by the horizontal white and black bars (LD); DD is indicated by gray and black bars. The total number of flies that eclosed during the experiments are indicated in parentheses for each genotype. Error bars represent standard errors of the binomial distributions.

The *E74* locus is known to be essential for ecdysis in *Drosophila* (reviewed in [43]–[45]). Loss-of-function alleles of *E74* cause a failure of ecdysis and thus lethality [46]. In our phenotypic screen, populations homozygous for *E74^BG01805^*, a viable insertion allele, displayed a striking early-eclosion phenotype, reminiscent of the phenotype reported for a strain with decreased *lark* gene dosage [47]. In the *E74^BG01805^* homozygous population, eclosion commenced just after the lights-off signal (ZT12)–many hours earlier than normal–when populations were entrained to a cycle consisting of 12 hours of light and 12 hours of dark (LD 12∶12). Reproducibly, the mutant eclosion profile was observed to have two peaks: a minor one at ZT16 and a major peak after the lights-on signal (between ZT 0 and 2; Figure 3A). In the mutant population, 54.3% of the adults eclosed prior to lights-on, compared to 21.5% in the control population (Figure 3E). A similarly abnormal pattern of eclosion was observed for the mutant in free-running (constant dark or DD) conditions following entrainment to LD 12∶12 (Figure 3C and 3E). Moreover, transheterozygous populations carrying *E74^BG01805^* in trans to a chromosome deletion uncovering the *E74* region [Df(3L)81k19, breakpoints 73A3; 74F1-4] displayed a severe eclosion phenotype, in which the majority of flies eclosed during the night portion of the cycle (Figure 3D and 3E). This result maps the factor causing the behavioral phenotype to the 73A3 to 74F1-4 genomic interval, which contains the *E74* locus, and suggests that *BG01805* is a hypomorphic allele of *E74*. Finally, excision of the P-element in the *E74^BG01805^* strain completely restored wild-type eclosion rhythms (Figure 3B and 3E), demonstrating that the P-element insertion causes the mutant phenotype.

The *E74^BG01805^* mutation has rhythm-specific effects on circadian periodicity; it dramatically alters the gating of eclosion but does not affect the daily phasing of locomotor activity (Figure 3F). Nevertheless, even after outcrosses to minimize genetic background differences between mutants and two different revertant strains (bearing excision chromosomes lacking the P element), we observed small, albeit statistically significant, effects of the *E74^BG01805^* mutation on the activity rhythm. Circadian period was slightly short for the mutant compared to one revertant line, whereas rhythmicity index (RI) was slightly decreased compared to either revertant (Table 1). However, these differences are extremely small (on the order of ∼15 min for circadian period) and unlikely to be biologically meaningful.

**Table 1:** Average rhythmicity index (RI) and period length for wild-type, E74^BG01805^ mutant and revertants.

Genotype	Average Rythmicity Index (RI)	Average Period (hour)
*w^1118^*	0.61±0.01	23.91±0.04
*E74^BG01805^*	0.46±0.02 *	23.66±0.06 **
*E74^R3^*	0.57±0.01	23.91±0.04
*E74^R6^*	0.52±0.02	23.80±0.04

* P<0.001 compared to *E74^R3^*, or P<0.05 compared to *E74^R6^* based on Student's t-test,

** P<0.001 compared to *E74^R3^* based on Student's t-test. n = 30 for *w^1118^*, n = 32 for *E74^BG01805^*, *E74^R3^*, and *E74^R6^*.

Consistent with a physical interaction between LARK and the *E74* transcript, in vivo, we observed a genetic interaction between *lark^1^* and the *E74^BG01805^* allele. In our studies, we found that *E74^BG01805^* homozygotes survive to adulthood, presumably because the mutant retains residual E74 function (null E74 mutations are lethal). In contrast, the *lark^1^* mutation is a recessive lethal, although most mutant homozygotes survive until the early pupal stage (94±2.5% in the present study). Interestingly, only 12±2.7% of the homozygous *lark^1^; E74^BG01805^* double mutants survived to the early pupal stage; i.e., most of them died prior to or at the third-instar larval stage, indicating that the *BG01805* mutation enhanced the lethal phenotype of *lark^1^*. This observation also indicates that LARK is not absolutely required for synthesis of E74A; i.e., there must be some E74 synthesis in the complete absence of LARK, as the phenotype of the *lark^1^* mutant becomes more severe with decreased E74 gene function.

**Figure 4:**
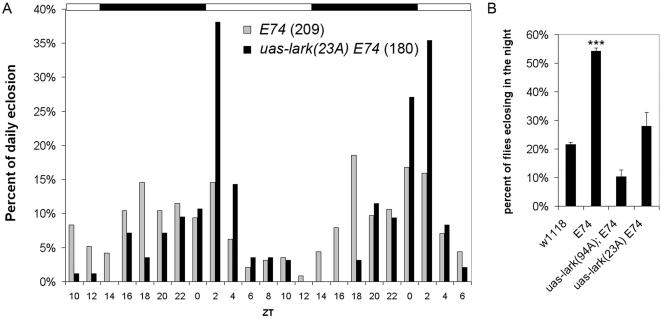
Overexpression of LARK suppresses the mutant eclosion phenotype of *E74^BG01805^* populations. A) Eclosion profiles of *E74^BG01805^* and *E74^BG01805^*, *UAS-lark^23A^* populations. The total number of flies that eclosed during the experiments are indicated in parentheses for each genotype. B) Percentages of flies eclosing between ZT10 and ZT22 are quantified for various genotypes. The data in panel B for the *E74* and *UAS-lark^23A^ E74* populations are from day one of the results shown in panel A. For panel B, n = 86 for *UAS-lark(23A)E74*. n = 183 for *UAS-lark(94A); E74*. n = 2544 for *w^1118^*. n = 1963 for *E74*. *** P<0.0001 compared to all other genotypes, based on Chi-sqare test for equality of distributions. Error bars represent standard error of the binomial distribution. E74 refers to *E74^BG01805^* ; 94A and 23A are two different independent strains carrying *UAS-lark* transgenes [28].

Perhaps more important, we found that overexpression of LARK suppresses the mutant phenotype of *E74^BG01805^*, as expected if LARK promotes E47 production. The *E74^BG01805^* mutation was generated by a “gene trap” insertion that includes a GAL4 coding segment downstream of the *E74* gene promoter in an orientation appropriate for expression under control of the promoter [48]. Thus, in *E74^BG01805^* mutants, GAL4 expression ought to be driven by the native promoter of the E74 gene. We introduced a *UAS-lark* transgene, genetically, into the *E74^BG01805^* background and examined eclosion in *E74^BG01805^* (*E74-Gal4*), *UAS-lark* and mutant control populations. We found that overexpression of LARK partially or fully suppressed night-time eclosion events for the *E74^BG01805^* mutant population on two consecutive days of LD (Figure 4).

**Figure 5:**
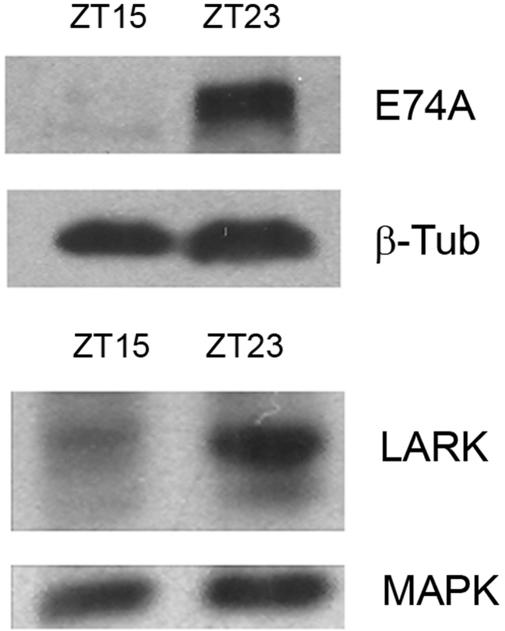
E74A protein shows diurnal changes in abundance. Western Blots show detection of the E74A (upper panel) and LARK (lower panel) proteins at ZT15 and ZT23 in pharate adults overexpressing LARK under control of the *elav-gal4* driver. Similar results were observed in two independent experiments.

Previous research found that LARK abundance oscillates in a circadian manner [25]. If LARK facilitates translation of E74A, as suggested by results shown in Figure 2, then E74A protein abundance might show diurnal changes in abundance, in phase with LARK. To test this hypothesis, we examined E74A abundance in pharate adults at ZT15 and ZT23, time-points at which LARK abundance differs (i.e., it is lower at ZT15). We found that E74A abundance was extremely low in wild-type flies at the pharate-adult stage, and thus we could not reliably compare levels at the two different times of day. However, the pan-neuronal overexpression of LARK dramatically increased E74A protein at this stage, and there were corresponding diurnal changes in abundance (Figure 5). We note that pan-neuronal overexpression of LARK, using this particular Gal4 driver (*elav-gal4*) increases LARK abundance without significantly altering the phase of the LARK oscillation (unpublished results). These results suggest that rhythmic changes in LARK abundance may drive rhythms in E74 levels *in vivo*.

## Discussion

### A biochemical genetic strategy to identify circadian targets of LARK

We have employed a microarray-based Ribonomics approach to define RNAs that are associated with LARK in vivo. This was followed by a phenotypic screen of relevant mutants to select functionally relevant targets from the candidate pool. Several lines of evidence suggest that many of the RNAs identified in our analysis represent *in vivo* targets of LARK. First, known targets of RBM4 (mLARK), a mammalian homolog of fly LARK, were identified in our studies. Second, most of the identified transcription units have features in common. Third, the overexpression of LARK caused increased abundance for two target-encoded proteins for which antisera are available. Fourth, consistent with a circadian role for LARK, target genes with circadian functions were identified. Lastly, we have documented genetic interactions between *lark* and one target gene (E74). Although direct binding of LARK to target RNAs has not been verified *in vitro*, the aggregate of our results suggests that many of these RNAs are biological targets of LARK *in vivo*.

### A large and diverse set of LARK target RNAs

Given that LARK is an essential protein with a broad expression pattern in the CNS and other tissues [49], it is not surprising that the protein interacts with a large and diverse set of RNA molecules. The protein may interact with distinct target RNAs in different cell types to regulate many cellular processes. As shown in Table S2, the putative target RNAs encode proteins that function in many different processes including neurotransmitter biosynthesis, synaptic transmission, membrane excitability, nuclear/cytoplasmic transport, signal transduction, cell adhesion, cell proliferation and cell death. This list of potential targets represents a good starting point for identifying LARK-regulated RNAs that function in diverse cellular processes.

Recent studies in other organisms have found that most cellular processes, including the cell cycle, are regulated by the circadian clock [50]–[52]. Taken together with the observation that LARK abundance shows diurnal changes in all neurons (V. Sundram and F. R. Jackson, unpublished results), a diverse set of LARK targets also suggests a broad circadian regulation of cellular events in the fruit fly and a critical role for LARK in regulating these diverse circadian outputs.

### How does LARK regulate expression of its target RNAs?

The RRM class of RNA binding proteins are known to function in post-transcriptional regulatory processes, including the control of RNA splicing, intracellular transport, stability and translation [30]. Interestingly, a mammalian homolog of LARK (mLARK or RBM4a) was found to be involved in splicing of its target RNAs [32], [53]. The observation that many of the transcription units expressing LARK target RNAs contain unusually large introns suggests that fly LARK might facilitate the splicing of such introns and thus regulate protein expression. Although the nuclear localization of LARK in most neurons suggests a role of LARK in splicing rather than translation, LARK is localized to the cytoplasm in subsets of neurons such as the Crustacean Cardioactive Peptide (CCAP) neurons in the ventral ganglia. In addition, it may be present at low abundance in the cytoplasm of all neurons. Furthermore, the protein probably shuttles between the nucleus and cytoplasm, similar to the behavior of mLARK [54], [55] to regulate translational events. We note that the majority of LARK target RNAs contain miRNA binding sites. Since miRNAs mediate repression of translation [34], it is possible that LARK functions with miRNAs to regulate the translation of certain targets, similar to that postulated for other RNA-binding proteins such as the Fragile×Mental Retardation Protein (FMRP, [56], [57]). The observation that LARK overexpression increases protein abundance for certain targets without altering RNA levels is also consistent with a role for LARK in the regulation of translation. Such a role was postulated for the mLARK protein in recent studies of the mammalian clock system [33]. Because increased LARK expression has only minimal effects on the steady-state levels of most of the putative targets, we think it is unlikely that the RNA-binding protein regulates stability for most of these mRNA molecules, although it may have such a function for a subset of targets.

### Functions for LARK and certain target RNAs in Circadian Timing

Previous studies have shown that decreased or increased LARK expression results in altered rhythmicity, with overexpression of the protein leading to arrhythmicity for both the eclosion and locomotor activity rhythms [28]. As PER protein cycling appears to be normal in flies with increased LARK expression, the current model for the circadian function of LARK is that it modulates clock outputs, rather than regulating the central molecular oscillator[25], [28]. We note, however, that a mammalian homolog of LARK (mLARK or RBM4a) has been reported to function in the translational regulation of *mPer1* clock RNA [33]. Although our studies have identified many potential LARK target RNAs, the fly *Per* RNA is not among the collection. Thus, at present there is no evidence that *Per* represents a target of LARK in the fly circadian system.

Our ongoing behavioral screen has identified mutants of several different targets that exhibit altered locomotor or eclosion rhythms (Y.H., M.A.R. and F.R.J., unpublished results), suggesting a model wherein LARK regulates different output pathways by interacting with distinct RNA targets. Of the identified LARK targets, we have characterized one–E74–in some detail. E74 is one of several early response genes that are induced directly by ecdysone, the steroid hormone triggering insect ecdysis (reviewed in [43]–[45]). *E74* encodes two transcription factor isoforms, E74A and E74B, which act to induce and repress, respectively, transcription of downstream genes, thus achieving a precise regulation of the timing of downstream responsive genes during ecdysis [58]. We have documented a circadian phenotype in an *E74* mutant, suggesting that one or both of the E74 isoforms serves to regulate the circadian timing of adult eclosion. Interestingly, the ecdysis triggering hormone (ETH) promoter contains an imperfect E74 binding site [59], suggesting that E74 may also function in the epitracheal system to regulate expression of ETH. We point out that a circadian role for ecdysone and ecdysone-responsive genes in the regulation of eclosion is consistent with studies in other insects that have demonstrated a circadian synthesis of ecdysone [60] and with studies in *Drosophila* which indicate that the prothoracic gland (which synthesizes and releases ecdysone) contains a PER-based oscillator [61] that is required for normal eclosion rhythms [62]. It is an intriguing possibility that timed ecdysone release results in the activation of *E74* expression in neurons relevant for rhythmicity and that LARK serves a post-transcriptional role within such neurons that further coordinates the temporal expression of E74 protein and the daily gating of eclosion events.

## Materials and Methods

### Immunoprecipitation of LARK-RNPs

Our methods were previously described in reference [29]. Pharate adult brains of Canton-S wild-type flies were dissected in Drosophila SFM media (GIBCO 10797-017). Aliquots containing approximately 100 brains were flash frozen in liquid nitrogen and stored at −80°C. Dissections were carried out during the day (ZT0–ZT10) when LARK abundance is high. In order to minimize variation introduced by RNA amplification, a relatively large amount of tissue was used for the preparation of brain lysates (∼1000 brains per immunoprecipitation experiment). At the time of the experiment, brains were thawed in 150 µl polysome lysis buffer[31]. The tissues were dispersed by grinding gently with a plastic pestle. Because LARK is a nuclear protein, a MicroSon Cell Disruptor (Model XL2005) was used to break nuclear membranes. The output of the sonicator was set to low and brief (several sec) pulses were employed to break membranes. Small aliquots of the lysate were removed after each sonication pulse, stained with DAPI (Vector laboratories H-1200) and examined by florescence microscopy to determine the integrity of nuclei. When most nuclei were broken, the lysate was centrifuged at 14000×g for 10 minutes and the supernatant was saved. Ten µl of the supernatant was saved for the isolation of a total RNA sample. The remaining lysate (∼100 µl) was employed for immunoprecipitation of LARK-RNPs using 100 µl of affinity-purified anti-LARK antibody [25].

These experiments were carried out according to standard procedures [31] with the following modifications: 1. We used a large amount of starting material, i.e. ∼1000 hand-dissected Drosophila brains in each immunoprecipitation experiment. This ensured that we started with a large amount of LARK-RNPs. 2. We used very mild washing conditions after the immunoprecipitation. As a consequence, the IP sample contained most RNA species present in the total RNA sample. This is reflected by the observation that the number of genes detected in the IP arrays was similar to that detected in the total RNA arrays. 3. Equal amounts of RNA from the IP sample and from the total RNA sample were used for labeling and hybridization to the microarrays. This, combined with point 2 above, ensured that we detected a similar number of genes on the IP and total RNA arrays (e.g., 1880 for the IP and 2171 for the total RNA samples used in experiment one). In addition, the signal intensities of the arrays were also very similar. Thus, we were able to use normalization methods designed for regular expression arrays.

### Gene microarray analysis

Labeled RNAs were generated using the Affymetrix one cycle cDNA synthesis and IVT labeling kit (Affymetrix 900493, 900449) and hybridized to Drosophila whole genome microarrays (Affymetrix 900335). Microarray hybridization, washing and scanning were carried out on an Affymetrix Fluidics Station 400 and an Agilent GeneArray Scanner according to standard protocols provided by Affymetrix, Inc. For the identification of potential LARK targets, equal amounts of RNA (about 3 ug) from the total RNA sample or the immunoprecipitated LARK-RNPs were labeled and hybridized to Drosophila Genome Arrays chips. Due to the large amount of starting material (∼1000 brains per experiment), and the mild washing conditions employed after the IP, a similar spectrum of RNA species was detected in the IP and total RNA arrays; this facilitated normalization using the total intensity of the arrays. Signals were scaled to the same target intensity for the IP and total RNA arrays. Data were analyzed using the Affymetrix Microarray Suite 5.0. The default settings were used for all tunable parameters (i.e. tau = 0.015, α1 = 0.04, α2 = 0.06, perturbation factor = 1.1). The following criteria were used to identify a differential expression between samples: first, it was required that the signal for a given gene be identified as “present” in the experimental array; although it was considered acceptable that the signal for the same gene in the baseline array was “marginal” or “absent”. The total number of genes identified as “present” in the IP array was 1880 for experiment one and 1885 for experiment two. Second, we required a change in p value of less than 0.001 for an increase or greater than 0.999 for a decrease. Finally, it was a requirement that the signal log ratio was greater than 1 for an increase or smaller than -1 for a decrease, either of which translates into a two-fold difference in signal intensity between the two samples.

For comparisons between LARK overexpression and control flies, total RNA samples were prepared from adult heads of *elav-gal4/+; UAS-lark*/+ flies and *elav-gal4*/+; +/+ flies, respectively. About 10 µg of total RNA from each sample was used for labeling and hybridization to one gene chip. Three independent labeling and hybridization experiments were performed for each genotype. Raw data were normalized using the GCRMA package. After normalization, a filter was applied to select for genes that had a raw expression value of greater than 40 on at least one of the arrays. The filtered data sets were then analyzed using the R statistical software package with Bioconductor. Linear models were used in assessing the differential expression between conditions [63].

### Sequence analysis of putative LARK targets

The Multiple Alignment Construction and Analysis Workbench (MACAW) program [64] was used to identify common patterns in the 3′UTRs of the putative targets. UTR sequences for all target genes were downloaded from Flybase (Release 5.1, September 2006). For genes with multiple 3′UTR sequences, the longest sequence was used to represent the mRNA. An “A-rich” sequence was identified by the MACAW program as present in the 3′UTRs of the majority of the LARK targets. Due to the limitation of MACAW in handling large sequence sets, a custom-developed computer program was used instead to scan for such “A-rich” sequences in the 3′UTRs of genes expressed in pharate adult brains (i.e. genes detected by the microarrays in the brain total RNA samples) and in 3′UTRs randomly sampled from the whole genome. This program identifies sequence elements that are at least 15 nucleotides long with at least 75% of the sequence containing “A” residues. The frequencies of such 3′UTR A-rich elements were then calculated for LARK targets, brain total RNAs, and randomly sampled RNAs from the whole genome.

A database of intron number and size for all intron-containing genes of Drosophila was kindly provided by Flybase. Percentages were calculated for genes that contain at least one intron bigger than 10kb or 40kb for the whole genome pool, the pool of genes that were detected in the brain total RNA samples and the pool of LARK targets. Chi-square tests were performed to determine differences in frequency distribution for given intron sizes between the different types of gene collections.

To calculate the frequency of consensus miRNA sequences within targets, genes containing an annotated 3′UTR longer than 30 bp (i.e., long enough for miRNA binding) were scanned using the Drosophila miRNA database constructed by Enright et al [36]. Searches were performed for the LARK targets identified in each experiment (94 genes for experiment one and 127 genes for experiment two) and for genes that were identified in the total RNA sample for each experiment (1954 genes for experiment one and 1490 genes for experiment two). Searches also were performed on all 9805 genes in the fly genome that contain a qualifying annotated 3′UTR.

### Quantification of RNA and protein amounts

Total RNA samples were prepared from adult fly heads of either *elav-gal4/+; UAS-lark*/+ or *elav-gal4*/+; +/+ populations and reverse-transcribed into cDNAs using Superscript II-RT (Invitrogen). Quantitative PCR was performed in a Strategene Maxpro×using the SYBR green method. Primers used for amplifying Vap33-1 specific fragments were: CCGGCCGTCAAACAGGTG and TGCCCAGCAGGAGGCTAACG. Primers used for amplifying E74 specific fragments were: GGAGCGAATGGACAAGCTCA and GCTGTTGCAGGTGGTGCT. Primers used for amplifying the Rp49 specific fragments were as previously described [65].

The Vap33-1 and E74A proteins were visualized by Western blotting. Antibody for fly Vap33-1 was obtained from Hugo Bellen [66]. Antibody for E74A was provided by Carl Thummel [67]. Mature pupae containing pharate adult flies of either the *elav-gal4/+; UAS-lark*/+ or *elav-gal4*/+; +/+ population were homogenized in polysome lysis buffer [31] and the supernatants were separated by SDS-PAGE electrophoresis. Approximately 120 ug of total cellular protein was loaded in each lane. Protein blotting was carried out according to standard protocols. The intensities of bands on the blots were quantified using a Kodak image station 440 with the Kodak 1D 3.5 software.

### Stocks and behavior genetic screens

Mutants of LARK targets were obtained from the Bloomington, Szeged and Harvard/Exelixis stock centers and from GenExel, Inc. (Daejeon, South Korea). A recombinant chromosome carrying the *E74^BG01805^* insertion and a *UAS-lark* transgene was generated using standard genetic techniques. To screen mutant populations for altered eclosion rhythms, fly stocks were maintained in vials at 18°C under a LD 12:12 schedule for at least 5 days prior to adult eclosion. At the time of the eclosion experiment, the vials were cleared of adult flies at around ZT 11 and then examined at approximately ZT 0 to identify those with significant night-time eclosion. If a relatively large number of flies were seen at ZT 0, then the mutant was designated as having an early-eclosion profile. The vials were cleared of adults again at ZT 6 and then examined at ZT 11 to determine if a large number of flies emerged late in the daily gate; such mutants were presumptively designated late-eclosion strains. To examine complete profiles of population eclosion, cultures were maintained at 18°C in LD 12∶12 for at least 5 days prior to the eclosion experiment. At the time of the eclosion experiment, emerging adults were collected every two hours under the same LD 12∶12 schedule or in constant darkness (DD). A lamp with a 7.5 watt bulb and Kodak GBX-2 filter provided red light in DD.

To screen for mutants with a defective locomotor activity rhythm and to further characterize new activity mutants, stocks were raised at 25°C in LD 12∶12. After eclosion, newly emerged male flies were loaded into glass tubes with medium and monitored using the Drosophila Activity Monitoring (DAM) system. Activity was recorded at 23°C in LD 12∶12 for 3–4 days and then in constant darkness (DD) for at least 7 days. Activity data were analyzed for circadian periodicity using the Fly Toolbox algorithms developed by Levine et al [68]. Period was determined by periodogram analysis and the robustness of rhythmicity was examined using the Rhythmicity Index (RI) function of this package.

## Supporting Information

Table S1 (0.04 MB XLS)

Table S2 (0.09 MB DOC)
