# Supplementary material for: The LARK RNA-Binding Protein Selectively Regulates the Circadian Eclosion Rhythm by Controlling E74 Protein Expression
Source: PLoS One. 2007 Oct 31;2(10):e1107. doi: 10.1371/journal.pone.0001107 (PMC2040218; doi:10.1371/journal.pone.0001107)
Supplement: Table S2 — (0.09 MB DOC) [file pone.0001107.s002.doc]

Supplemental Table 2. Functional classification of the 79 LARK targets common

to both immunoprecipitation experiments.

| **FlyBase ID** | **Symbol** | **Function** |
| --- | --- | --- |
| FBgn0027885 | Aac11 | Apoptosis |
| FBgn0000303 | Cha | Biosynthesis, acetylcholine |
| FBgn0038641 | CG7708 | Biosynthesis, acetylcholine |
| FBgn0027291 | l(1)G0156 | Biosynthesis, amino acid |
| FBgn0039358 | CG5028 | Biosynthesis, amino acid |
| FBgn0003423 | slgA | Biosynthesis, glutamate |
| FBgn0010415 | Sdc | Cell adhesion |
| FBgn0000547 | Ed | Cell adhesion |
| FBgn0024753 | Flo-2 | Cell adhesion |
| FBgn0015774 | NetB | Cell adhesion |
| FBgn0011661 | Moe | Cytoskeleton |
| FBgn0051363 | Jupiter | Cytoskeleton associated |
| FBgn0001186 | Hex-A | Glycolysis |
| FBgn0000064 | Ald | Glycolysis |
| FBgn0041094 | Scyl | Growth |
| FBgn0029761 | SK | Ion channel, potassium channel |
| FBgn0028704 | Nckx30C | Ion channel, calcium channel |
| FBgn0002921 | Atpalpha | Ion channel |
| FBgn0004244 | Rdl | Ion channel |
| FBgn0036043 | CG8177 | Ion channel |
| FBgn0036642 | CG4169 | Mitochondrial |
| FBgn0020497 | Emb | Nuclear/cytoplasmic transport |
| FBgn0024921 | Trn | Nuclear/cytoplasmic transport |
| FBgn0000986 | Fs(2)Ket | Nuclear/cytoplasmic transport |
| FBgn0003498 | Sqd | Nuclear/cytoplasmic transport |
| FBgn0029896 | CG3168 | Nucleic acid binding |
| FBgn0035720 | CG10077 | Nucleic acid binding |
| FBgn0052647 | CG32647 | Nucleic acid binding |
| FBgn0019890 | Smg5 | Nucleic acid binding |
| FBgn0010110 | East | Proteolysis |
| FBgn0031057 | CG14224 | Proteolysis |
| FBgn0020611 | Vha44 | Proton transport |
| FBgn0030897 | Frq1 | Signaling, calcium-mediated pathway |
| FBgn0004625 | norpA | Signaling, calcium-mediated pathway |
| FBgn0000253 | Cam | Signaling, calcium-mediated pathway |
| FBgn0017549 | Ric | Signaling, G protein-mediated pathway |
| FBgn0001122 | G-oalpha47A | Signaling, G protein-mediated pathway |
| FBgn0014011 | Rac2 | Signaling, G protein-mediated pathway |
| FBgn0040335 | CG10260 | Signaling, kinase |
| FBgn0033791 | Drl-2 | Signaling, kinase |
| FBgn0015380 | Drl | Signaling, kinase |
| FBgn0031294 | ia2 | Signaling, phosphatase |
| FBgn0000479 | Dnc | Signaling, phosphodieserase |
| FBgn0004907 | 14-3-3zeta | Signaling, PKC inhibitor |
| FBgn0024963 | GluClalpha | Synaptic transmission |
| FBgn0029687 | Vap-33-1 | Synaptic transmission |
| FBgn0038947 | Sar1 | Synaptic transmission |
| FBgn0053528 | CG33528 | Synaptic transmission |
| FBgn0013334 | Sap47 | Synaptic transmission |
| FBgn0008654 | Su(z)2 | Transcription factor |
| FBgn0011481 | Ssdp | Transcription factor |
| FBgn0031759 | Lid | Transcription factor |
| FBgn0026160 | Tna | Transcription factor |
| FBgn0053100 | 4EHP | Translation |
| FBgn0037686 | RpL34b | Translation |
| FBgn0026250 | eIF-1A | Translation |
| FBgn0010488 | l(2)01424 | Translation |
| FBgn0037615 | CG11760 | Unknown |
| FBgn0052714 | CG32714 | Unknown |
| FBgn0030090 | ld14 | Unknown |
| FBgn0030981 | CG14191 | Unknown |
| FBgn0031453 | CG9894 | Unknown |
| FBgn0035882 | CG13666 | Unknown |
| FBgn0039381 | CG17370 | Unknown |
| FBgn0039843 | CG15567 | Unknown |
| FBgn0040587 | CG17618 | Unknown |
| FBgn0053516 | Dpr3 | Unknown |
| FBgn0021800 | l(2)k16918 | Unknown |
| FBgn0027581 | CG6191 | Unknown |
| FBgn0035495 | CG14989 | Unknown |
| FBgn0039647 | CG14509 | Unknown |
| FBgn0027542 | CG6014 | Unknown |
| FBgn0028480 | CG17841 | Unknown |
| FBgn0036773 | CG13698 | Unknown |
| FBgn0052264 | CG32264 | Unknown |
| FBgn0040896 | CG14775 | Unknown |
| FBgn0026578 | CG7832 | Unknown |
| FBgn0051992 | CG31992 | Unknown |
| FBgn0037636 | CG9821 | Unknown |
